# Double Spherulite Formation via Two-Step Crystallization in PTT/PET Blends

**DOI:** 10.3390/polym16233357

**Published:** 2024-11-29

**Authors:** Kousuke Sugeno, Hiromu Saito

**Affiliations:** Department of Organic and Polymer Materials Chemistry, Tokyo University of Agriculture and Technology, Koganei-shi, Tokyo 184-8588, Japan; s228409z@st.go.tuat.ac.jp

**Keywords:** PTT, PET, blend, crystallization, double spherulite

## Abstract

We investigated the crystallization kinetics and morphology evolution of miscible crystalline/crystalline blends of poly(trimethylene terephthalate) (PTT) and poly(ethylene terephthalate) (PET) during isothermal melt crystallization. The integrated light-scattering intensity and the spherulite size increased gradually and then steeply as crystallization progressed in 70/30 PTT/PET at 215 °C, indicating the two-step crystallization behavior. The compact PET spherulite grew in the first step, and the dendritic PTT spherulite grew in the second step, forming the double spherulite consisting of a PET component in the inner region and a PTT one in the outer region. The spherulite size of PET increased nonlinearly with time, suggesting the exclusion of PTT from the crystal growth front. Atomic force microscopy (AFM) observation revealed that the PTT fibrils were interfiled within the PET spherulite in the inner region and continued outward to the outer region consisting of the PTT spherulite. These results suggest that the excluded PTT crystallizes into fibrils by interfiling crystallization within the inner PET spherulite, and then the interfiled PTT fibrils continue to grow outward to form the outer dendritic PTT spherulite after the spherulite growth of PET stops due to the excluded PTT at the growth front.

## 1. Introduction

Polymer blends are mixtures of different polymers. Many polymer blends contain crystalline polymers. Crystalline polymer blends are extensively studied because of the scientific interest in their unique crystalline morphology and the characteristic kinetics of crystallization [1,2,3,4,5,6]. As the component polymer crystallizes, the noncrystalline component is excluded from the crystal growth front [7,8,9,10,11,12,13,14,15,16,17,18,19,20,21]. The degree of exclusion increases with increasing diffusion of the noncrystalline component, while the crystal growth rate decreases [10,18,20]. When the noncrystalline component is excluded into the amorphous region between fibrils, the spherulite morphology becomes coarse and open [7,8,11,13,14,15,16,22,23,24]. As the degree of exclusion increases during spherulite growth, the concentration of the excluded component at the growth front of the spherulite increases, causing the spherulite growth rate to decrease with time, i.e., the spherulite size increases nonlinearly with time [14,22,23,24,25,26,27,28,29].

Due to the increasing degree of exclusion during spherulite growth, the spherulite in the inner region is ordered, while that in the outer region is disordered, with large amorphous pockets of noncrystallizable components, as observed in crystalline/noncrystalline polymer blends of poly(vinylidene fluoride) (PVDF) and poly(methyl methacrylate) (PMMA) [24]. The resulting morphology is a double spherulite with a double growth front in one spherulite. The double spherulite is one of the unique spherulite morphologies obtainable in crystalline polymer blends. In poly(ethylene oxide) (PEO)/PMMA blends, double spherulites are obtained through liquid–liquid phase separation at the spherulite growth front of PEO and propagation outward with convective concentration waves from the spherulite growth front of PEO [30]. Double spherulites can also be obtained via the α-to-γ′ crystal transformation of PVDF in PVDF/PMMA blends; i.e., banded α-spherulites are transformed into banded γ′-spherulites during the growth of the α-spherulites, and then, the growth of non-banded γ-spherulites is induced from the γ′-spherulites in the outer region of the banded γ′-spherulites [31].

In crystalline/crystalline polymer blends, there are a few examples of double spherulites formed via isothermal melt crystallization. When the melting temperatures, *T*_m_s, of the component polymers are close, double spherulites are formed at specific temperatures and blend contents, as observed in poly(L-lactic acid) (PLLA)/poly(oxymethylene) (POM) blends [32] and poly(butylene adipate) (PBA)/PEO blends [33]. For example, when 97/3 PLLA/POM undergoes isothermal crystallization at 145 °C, the PLLA crystallizes into compact spherulites in the inner region, and the POM spherulite grows outward from the growth front of the PLLA spherulite due to the accumulation of the excluded POM [32]. Double spherulites have also been observed in poly(ethylene succinate) (PES)/PEO blends with a large *T*_m_ difference of 36 °C, and two spherulite growth fronts of PES and PEO have been observed in one spherulite [34]. Double spherulites are also referred to as concentric spherulites [30], wagon-wheel spherulites [31], core–shell spherulites [32], and blended spherulites [34]. As described above, double spherulites can be formed using different processes depending on the crystallization rates of the component polymers, i.e., the component polymer with a higher crystallization rate tends to form the inner region of double spherulites, while the one with a lower crystallization rate tends to form the outer region. However, the details of the evolution of double spherulites are still unclear, although various crystallization behaviors have been proposed to occur in crystalline/crystalline polymer blends [35]. These proposed behaviors include interpenetrating crystallization, in which the spherulites of polymer A intrude into those of polymer B [32,33,34,36,37,38,39,40,41,42,43,44]; interlocking crystallization, in which the spherulites of polymer A grow from the inside of the spherulite of polymer B [45,46]; and interfiling crystallization, in which the crystallization of polymer A occurs within the spherulite of polymer B [32,47,48].

Recently, through a series of crystallization kinetic studies, we found that a double spherulite was developed during isothermal melt crystallization in a miscible crystalline/crystalline blend of poly(trimethylene terephthalate) (PTT) and poly(ethylene terephthalate) (PET) at a specific temperature and blend content, in which both PTT and PET can crystallize, and the crystallization rate of PET is faster than that of PTT [49]. In this study, in order to understand the origin of the double spherulite, we investigated the crystallization kinetics and morphology evolution of PTT/PET blends with polarized and optical microscopic observation, light-scattering measurement, atomic force microscopy (AFM) observation, and differential scanning calorimetry (DSC) measurement. These results are discussed on the basis of the exclusion behavior of the component polymer from the spherulite growth front and the interfiling crystallization.

## 2. Materials and Methods

### 2.1. Materials

Poly(trimethylene terephthalate) (PTT) was provided by Asahi Chemical Co., Ltd. (Tokyo, Japan). Poly(ethylene terephthalate) (PET) was provided by Teijin Limited (Tokyo, Japan). The melting temperatures, *T*_m_s, of PTT and PET are 228 °C and 258 °C, respectively.

### 2.2. Preparation of PTT/PET Blends

PTT and PET were melt-blended at 300 °C in an Imoto IMC-18D7 mixing chamber (Imoto machinery Co., Ltd., Kyoto, Japan) at a rotor speed of 200 rpm for 5 min. Prior to the melt-blending, the PTT and PET were dried in a vacuum oven at 100 °C for 24 h to prevent transesterification during melt-blending. To obtain a film specimen approximately 100 μm thick, the blend specimen was compression-molded at 300 °C for optical microscopy observation, light-scattering measurement, and differential scanning calorimetry (DSC) measurement. 

For atomic force microscopy (AFM) observation, a thin cast film approximately 10 µm thick was prepared using the solution mixing method. PTT and PET were dissolved in 1,1,1,3,3,3-hexafluoro-2-propano at 5% weight concentration. The solution was dripped onto a cover glass and smoothed and dried using an OSP-52-L60 wireless bar coater (OSG system products Co., Ltd., Aichi, Japan). The resulting thin film was dried under a reduced pressure of 10^−4^ mmHg at room temperature for 30 min.

After the isothermal melt crystallization, the melt-crystallized specimen was quenched in ice water to freeze the structure for DSC measurement and AFM observation.

### 2.3. Characterization

#### 2.3.1. Polarizing Optical Microscope Observation

Spherical growth during isothermal melt crystallization was observed using an Olympus BX53 polarizing optical microscope (Olympus Corp., Tokyo, Japan) equipped with a sensitive tint plate (optical path difference of 530 nm) and an Olympus DP74 digital camera (Olympus Corp., Tokyo, Japan). For isothermal melt crystallization, the film specimen was annealed at the desired crystallization temperature on a Mettler Toledo HS82 hot stage (Mettler Toledo, Columbus, OH, USA) mounted on the stage of the polarizing optical microscope. The changes in the morphology of the melt-crystallized specimen after heating were also observed with the polarizing optical microscope described above. The spherulite morphology was also observed with the optical microscope without crossed polarizers.

#### 2.3.2. Small-Angle Light-Scattering Measurement

A polarized He–Ne laser with a wavelength of 632.8 nm was used for the small-angle light-scattering measurement. The laser was applied vertically to the film specimen on a Mettler Toledo HS82 hot stage (Mettler Toledo, Columbus, USA) for isothermal melt crystallization. The scattered light was passed through the analyzer and then onto a high-sensitivity pco 1600 Charge-Coupled Device (CCD) camera (Tokyo Instruments, Inc., Tokyo, Japan). The optical axis of the analyzer was perpendicular to that of the polarized laser, i.e., Hv geometry. The two-dimensional scattering intensity data captured with the CCD camera at 800 × 600 pixels were obtained as digital data.

#### 2.3.3. DSC Measurement

DSC measurement for the melt-crystallized specimen (approximately 3 mg) was performed with a DSC-Q200 (TA Instruments, New Castle, DE, USA) at a heating rate of 10 °C/min under a nitrogen atmosphere.

#### 2.3.4. AFM Observation

AFM observation of the melt-crystallized specimen was performed with a Shimadzu SPM-9700HT scanning probe microscope (Shimadzu Crop., Kyoto, Japan). The height image was obtained by mapping the displacement of the cantilever on the surface of the specimen with a spring constant of 9 N/m and a resonance frequency of 150 kHz using an OMCL-AC200TS-C3 silicone cantilever (Olympus Corp., Tokyo, Japan).

## 3. Results and Discussion

### 3.1. Spherulite Morphology

**Figure 1:**
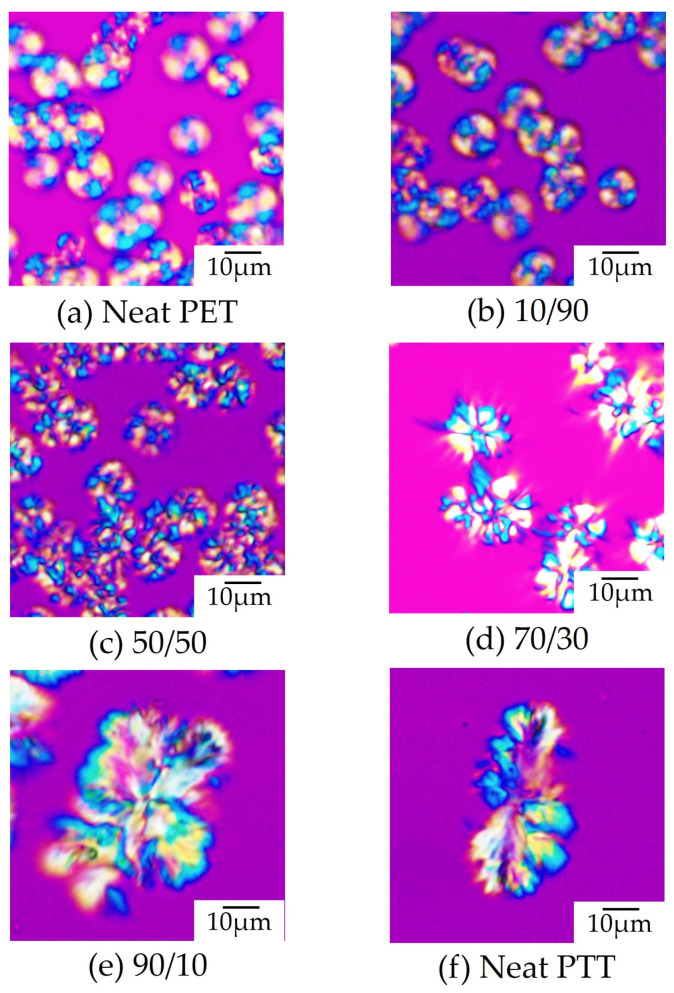
Polarized optical micrographs of neat PET, neat PTT, and PTT/PET blends with different contents observed during isothermal melt crystallization at 215 °C at different times: (**a**) 0/100 at 3 s, (**b**) 10/90 at 30 s, (**c**) 50/50 at 170 s, (**d**) 70/30 at 1800 s, (**e**) 90/10 at 1800 s, and (**f**) 100/0 at 600 s.

Figure 1 shows the spherulite morphology of poly(trimethylene terephthalate) (PTT)/poly(ethylene terephthalate) (PET) blends of different contents, as observed using polarized optical microscopy during isothermal melt crystallization at 215 °C, in which both PTT and PET can crystallize, and the crystallization rate of PET is faster than that of PTT. The spherulite morphologies of the neat PET and neat PTT are also shown for comparison. Compact spherulites with yellow and blue interference colors were formed in the neat PET due to the radial arrangement of the crystallites (Figure 1a), while large dendritic spherulites consisting of thick fibrils with a large cross-section several micrometers in size were formed in the neat PTT (Figure 1f). Compact spherulites observed in the neat PET were formed in the 20/80 and 50/50 PTT/PET blends (Figure 1b,c), while dendritic spherulites observed in the neat PTT were formed in the 80/20 blend (Figure 1e). In the 70/30 PTT/PET blend, both compact spherulites and dendritic spherulites were observed, indicating that PET spherulites and PTT spherulites can coexist (Figure 1d). These results indicate that PET spherulites grew in the blends with a PTT content of less than 90 wt%, while PTT spherulites grew in the blends with a PTT content of 70 wt% or more.

### 3.2. Two-Step Crystallization Behavior

Light-scattering measurement provides an overview of spherulite growth behavior. To estimate the crystallization kinetics, the integrated scattering intensity of the Hv geometry, in which the optical axis of the analyzer was perpendicular to that of the polarized laser, *Q*_Hv_, was used as follows:

(1) $$Q_{Hv}=\int_{0}^{\infty }I\left(q\right)q^{2}dq$$

where *I*(*q*) is the light-scattering intensity at *q*; *q* is the scattering vector; and $q=\left(4\pi /\lambda \right)\sin\left(\theta /2\right)$, *λ* and *θ* are the wavelength and scattering angle, respectively. *Q*_Hv_ describes the volume fraction of spherulite *ϕ*_s_ as follows:

(2) $$Q_{Hv}\propto {\phi_{s}\;\left(\alpha_{r}-\alpha_{t}\right)}^{2}$$

where *α*_*r*_ and *α*_*t*_ are the radial and tangential polarizability of the spherulite, respectively. Therefore, *Q*_Hv_ increases as *ϕ*_s_ increases during spherulite growth, and this increase stops when the growth stops.

**Figure 2:**
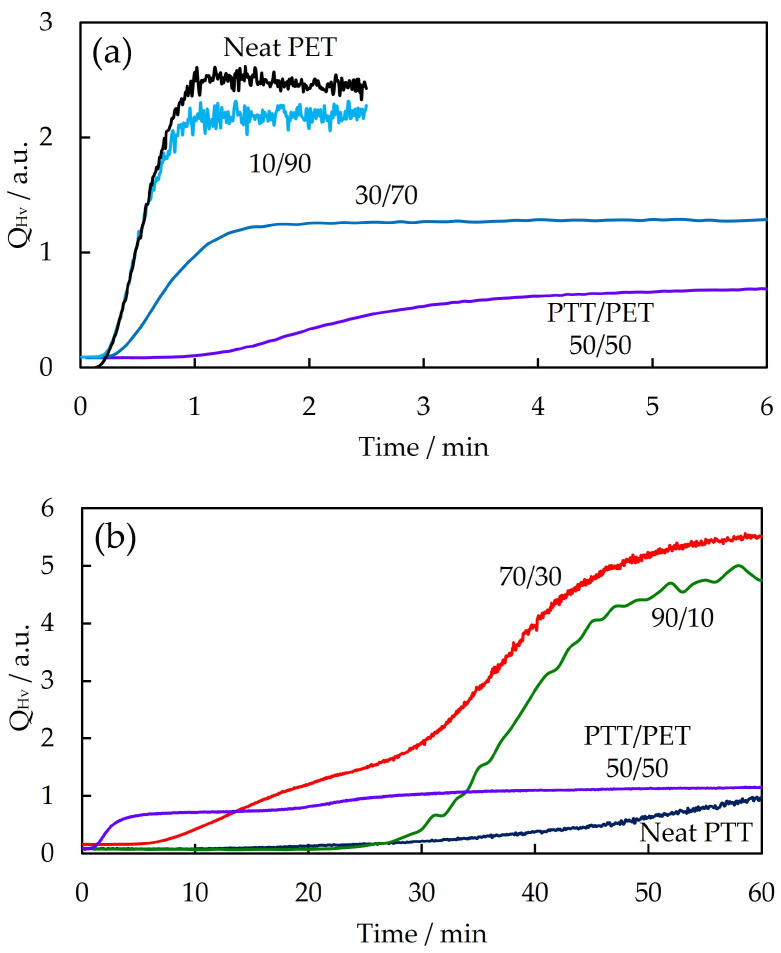
Time evolution of the integrated Hv light-scattering intensity, *Q*_Hv_, for PTT/PET blends with different contents during isothermal melt crystallization at 215 °C: (**a**) 0/100–50/50 PTT/PET, (**b**) 50/50–100/0 PTT/PET.

Figure 2 shows the time evolution of the integrated Hv light-scattering intensity *Q*_Hv_ for PTT/PET blends of different contents during isothermal melt crystallization at 215 °C. As expected from Equation (2), *Q*_Hv_ increased continuously with time and then stopped at 0/100, 10/90, 30/70, 50/50, 90/10, and 100/0 PTT/PET. On the other hand, a two-step increase was observed in 70/30 PTT/PET, i.e., *Q*_Hv_ changed continuously with two inflection points. *Q*_Hv_ increased gradually and then increased steeply with time in 70/30 PTT/PET. The increase in the second crystallization step was higher than that in the first crystallization step. The two-step increase in *Q*_Hv_ and the large increase in the second crystallization step observed in 70/30 PTT/PET differ from the continuous increase in *Q*_Hv_, which is usually observed in neat crystalline polymers and blends.

**Figure 3:**
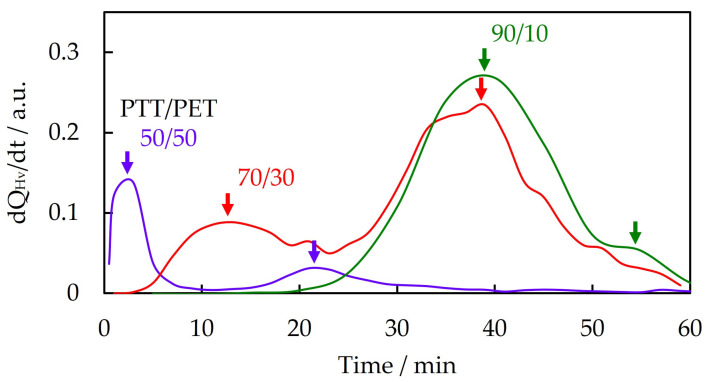
Time evolution of the derivative of *Q*_Hv_, d*Q*_Hv_/d*t*, for PTT/PET blends with different contents during isothermal melt crystallization at 215 °C. The peak is indicated by the arrow.

The two-step increase in 70/30 PTT/PET was confirmed by the appearance of two peaks in the derivative of *Q*_Hv_, d*Q*_Hv_/d*t*, versus time *t*, shown in Figure 3. The peak observed around 10 min is attributed to the first step increase, and the peak observed at around 40 min is attributed to the second step increase in *Q*_Hv_. The second peak was higher than the first peak, indicating that the increase in the volume fraction of the spherulite in the second crystallization step was greater than that in the first crystallization step due to the higher spherulite growth rate in the second crystallization step. Two peaks were also observed in 50/50 and 90/10 PTT/PET, indicating a two-step increase in *Q*_Hv_, but the second peak was much lower than the first peak.

**Figure 4:**
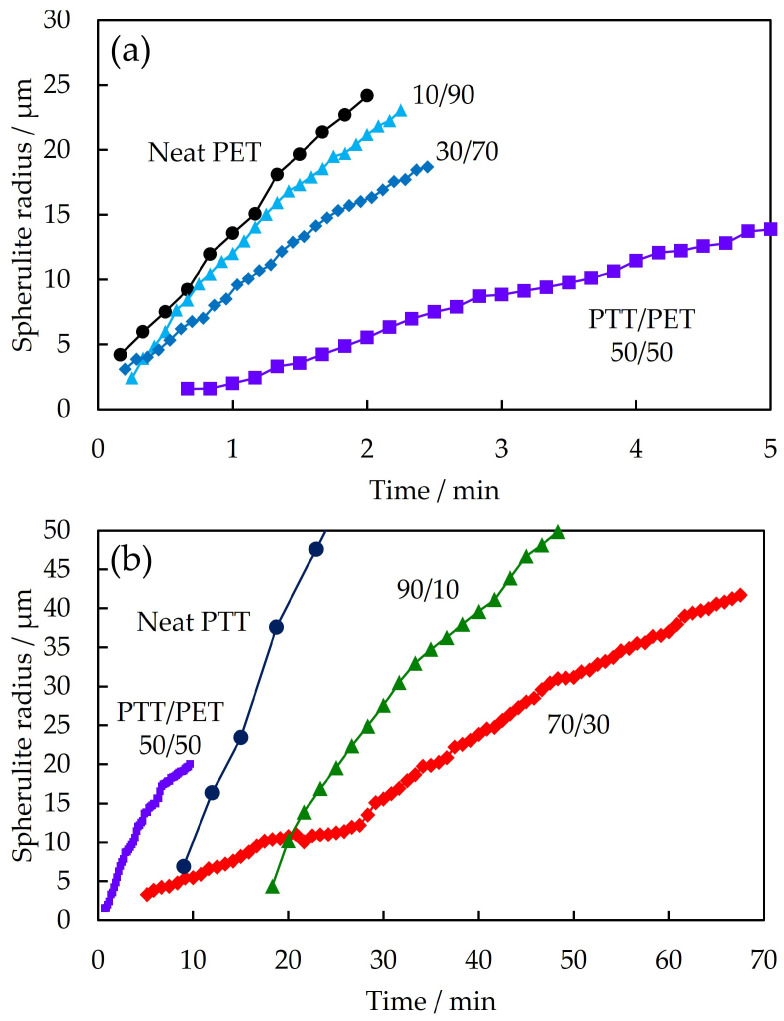
Time variation of the spherulite radius for PTT/PET blends of different contents during isothermal melt crystallization at 215 °C: (**a**) 0/100–50/50 PTT/PET, (**b**) 50/50–100/0 PTT/PET.

To understand the two-step increase in *Q*_Hv_ shown in Figure 2 and Figure 3, the change in spherulite radius during spherulite growth was determined from the polarized optical micrographs shown in Figure 1. Figure 4 shows the time variation of the spherulite radius for the different PTT/PET blends during isothermal melt crystallization at 215 °C. The slope of the radius versus time in Figure 4 depicts the spherulite growth rate, showing that the growth rate was faster when the slope was steeper. The spherulite growth rate of PET decreased when blended with PTT, and it decreased continuously with an increase in PTT content up to 70% (10/90–70/30 PTT/PET in Figure 2a,b). The spherulite growth rate was also delayed in the blend with a high PTT content, i.e., the spherulite growth rate of PTT decreased with an increase in PET content up to 30% (90/10 and 70/30 PTT/PET in Figure 2b). Nonlinear spherulite growth with time, i.e., a decrease in the spherulite growth rate with time, was observed in the blends, whereas linear growth was observed in the neat component polymers. The nonlinear growth was caused by the exclusion of the noncrystallizable component; i.e., the amount of excluded noncrystallizable components at the growth front increased with time during spherulite growth, as shown in our previous work [49].

The most interesting result, shown in Figure 4, is the two-step spherulite growth behavior observed in the 70/30 PTT/PET, i.e., the spherulite radius increased nonlinearly with time up to 30 min in the first crystallization step, and then it increased steeply in the second crystallization step (Figure 4b). Characteristically, the spherulite growth rate in the second crystallization step was faster than that in the first crystallization step, i.e., the spherulite growth rate increased during the second crystallization step. The two-step spherulite growth observed in Figure 4 is consistent with the two-step increase in *Q*_Hv_ shown in Figure 2. In nonlinear spherulite growth, which is commonly observed, the spherulite growth rate decreases continuously with time, and the growth rate never increases again after decreasing [5,6,14,22,24,26,27,29]. To the best of our knowledge, this study is the first to report this characteristic two-step spherulite growth behavior for crystalline polymer blends.

### 3.3. Evolution of Double Spherulite

**Figure 5:**
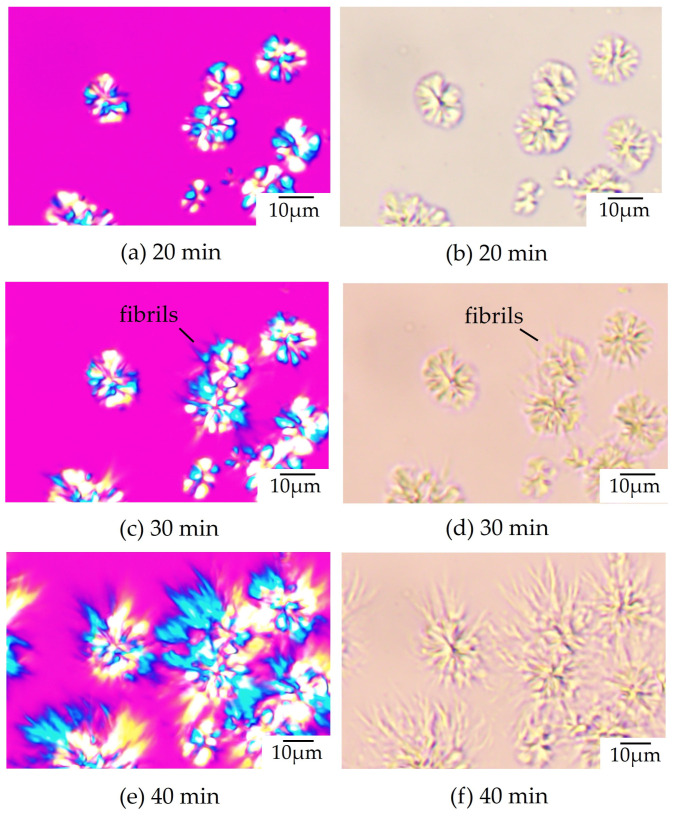
Morphology evolution of 70/30 PTT/PET during isothermal melt crystallization at 215 °C, observed with polarized optical microscopy (**a**,**c**,**e**) and optical microscopy (**b**,**d**,**f**).

To understand the characteristic two-step spherulite growth behavior observed in 70/30 PTT/PET (Figure 2b and Figure 4b), the evolution of the spherulite’s morphology during its growth, as observed with optical and polarized optical microscopy, is shown in Figure 5. Compact spherulites with yellow and blue interference colors were formed at an early stage of crystallization (Figure 5a). The compact spherulites were similar to the PET spherulites shown in Figure 1a, but they were coarser than those of the neat PET due to the presence of the excluded components between the fibrils, as indicated by the radially arranged black lines in the spherulite (Figure 5b). After the growth of the compact spherulites slowed, thick fibrils appeared on the surface of the compact spherulites between 25 and 35 min (Figure 5c), and thick fibrils from the spherulite growth front of PET were seen (Figure 5d). The compact spherulites transformed into dendritic spherulites (Figure 5e) as the thick fibrils grew into longer fibrils (Figure 5f). During the growth of the thick fibrils outside of the PET spherulite, the spherulite growth front of the PET became unclear. The dendritic spherulites were similar to the PTT spherulites shown in Figure 1f. Therefore, the two-step spherulite growth shown in Figure 2 and Figure 4 is attributed to the change in spherulite growth from a compact PET spherulite in the first crystallization step to a dendritic PTT spherulite in the second crystallization step.

**Figure 6:**
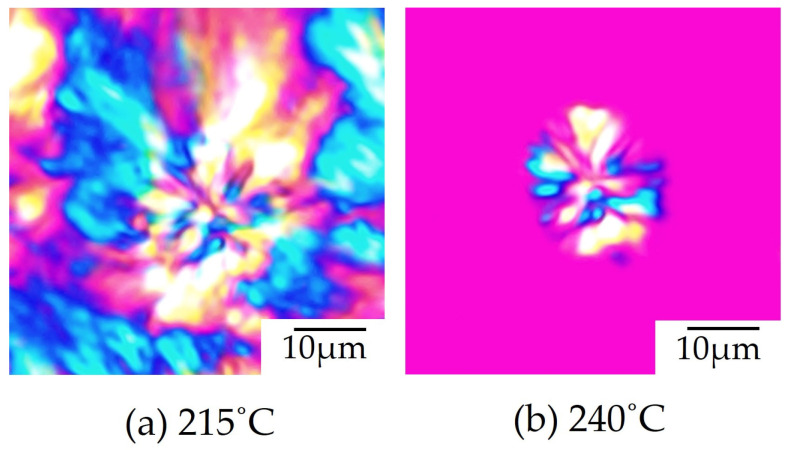
Morphology changes occurring during heating for the spherulites of 70/30 PTT/PET: (**a**) melt-crystallized specimen at 215 °C; (**b**) specimen heated up to 240 °C, which is above the melting temperature of PTT.

Figure 6 shows the change in morphology that occurred when heating the spherulites of 70/30 PTT/PET obtained via the two-step spherulite growth process shown in Figure 5. When the resulting volume-filled spherulites (Figure 6a) were heated to a temperature above the melting temperature of PTT, the dendritic PTT spherulite in the outer region obtained in the second crystallization step disappeared, while the compact PET spherulites in the inner region obtained in the first crystallization step remained (Figure 6b), as observed for the double spherulite of poly(L-lactic acid) (PLLA)/poly(oxymethylene) (POM) blends consisting of PLLA in the inner region and POM in the outer region [32]. Thus, a double spherulite was obtained via two-step spherulite growth from the compact PET spherulites in the inner region in the first crystallization step to the dendritic PTT spherulites in the outer region in the second crystallization step.

### 3.4. Exclusion Behavior

**Figure 7:**
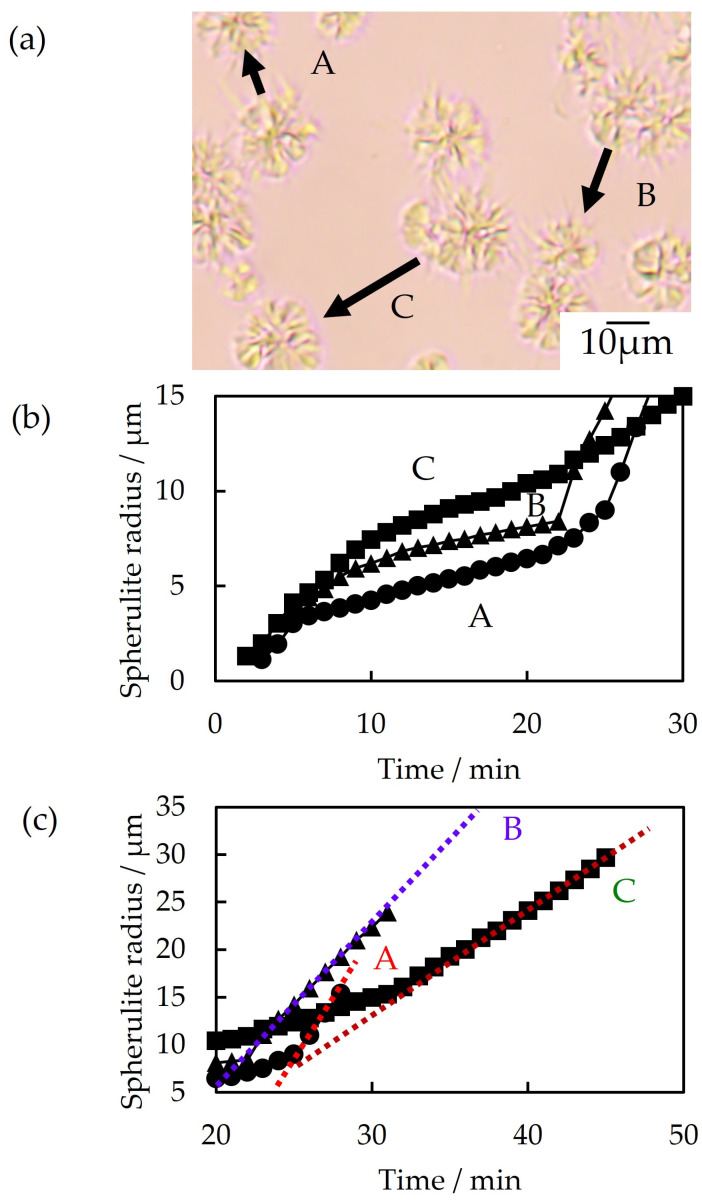
Spherulite growth with different interspherulite distances of 70/30 PTT/PET during isothermal melt crystallization at 215 °C: (**a**) optical micrographs of spherulites with different interspherulite distances, (**b**) time variation of the spherulite radius in the first crystallization step, and (**c**) time variation of the spherulite radius in the second crystallization step. Interspherulite distances: (A) 10 μm, (B) 20 μm, and (C) 30 μm.

As shown in Figure 7, the spherulite growth of 70/30 PTT/PET shown in Figure 4b varied with interspherulite distance. The spherulite growth rate of PET decreased in the first crystallization step at a shorter interspherulite distance, with the decrease becoming larger as the interspherulite distance became shorter. The spherulites, in order of shortest to longest, were A, B, and C (Figure 7a,b). The decrease in the growth rate is attributed to the interference of the PTT excluded in the interspherulite melt region between the PET spherulites, as has been reported for polypropylene/liquid paraffin blends [26]. As the interspherulite distance became shorter, the PTT concentration at the spherulite growth front of PET became higher, and the spherulite growth rate of PTT in the second crystallization step was faster, i.e., the spherulite growth rate in the second crystallization step for spherulites A, B, and C, as determined from the slope in Figure 7c, were 2.1, 1.7, and 1.0 μm/min, respectively. Thus, the spherulite growth in the second crystallization step was caused by the PTT being excluded from the growth front of PET in the first crystallization step.

**Figure 8:**
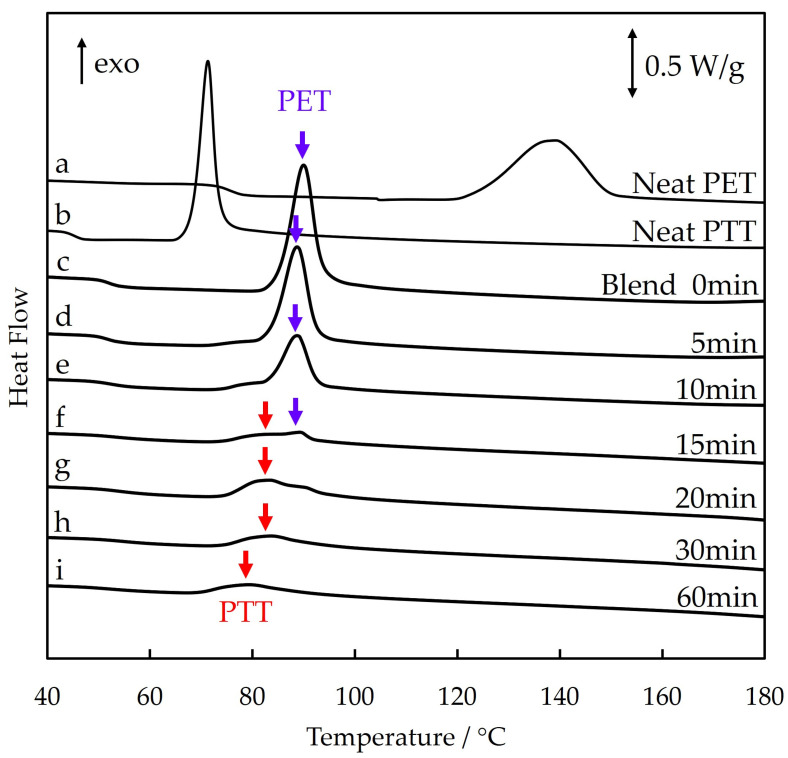
DSC thermograms for the heating process of 70/30 PTT/PET obtained via quenching after isothermal crystallization at 215 °C for different crystallization times (**a**–**i**).

Figure 8 shows the differential scanning calorimetry (DSC) thermograms for the heating process of 70/30 PTT/PET during isothermal melt crystallization at 215 °C for different crystallization times. The DSC thermograms of neat PET and neat PTT are also shown for comparison. The crystallization temperatures of neat PET and neat PTT during the heating process were 140 °C and 70 °C, respectively (Figure 8a and b). In the blend, an exothermic peak appeared at about 90 °C. This peak is attributed to the crystallization of PET. The shift of the peak to a lower temperature could be attributed to the accelerated crystallization induced by liquid–liquid phase separation during the heating process due to the existence of an upper critical solution-type phase diagram [50]. The area of the peak at about 90 °C decreased with time during crystallization as a result of the decrease in the crystallizable amorphous region of PET, which was due to the spherulite growth of PET in the first crystallization step (Figure 8c–f). After 15 min of crystallization, an exothermic peak appeared at about 80 °C (Figure 8f). This peak is attributed to the crystallization of PTT. The shift of the peak to a higher temperature could be attributed to the delay in crystallization due to the presence of PET in the excluded PTT, i.e., the excluded PTT on the outside of the PET spherulite was not pure PTT but mixed with PET. As a result of the presence of PET in the excluded PTT, the dendritic spherulite in the blend was more open than that of the neat PTT (Figure 1 and Figure 5), which was due to the exclusion of PET from the spherulite growth front of PTT in the second crystallization step. The area of the peak at around 80 °C during the crystallization of PTT decreased with crystallization time as a result of the decrease in the crystallizable amorphous region of PTT, which was due to the spherulite growth of PTT in the second crystallization step (Figure 8g–i). This result supports the crystallization of the excluded PTT in the second crystallization step.

### 3.5. Interfiling Crystallization

**Figure 9:**
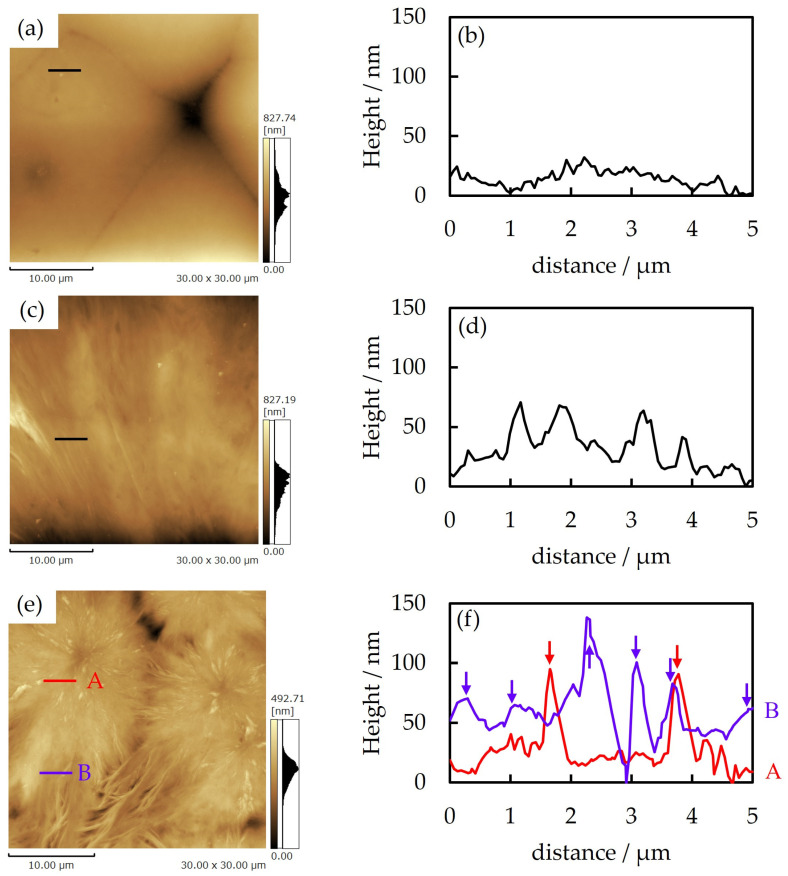
AFM height images and height profiles of neat PET, neat PTT, and 70/30 PTT/PET obtained through isothermal melt crystallization at 215 °C: (**a**,**b**) neat PET, (**c**,**d**) neat PTT, and (**e**,**f**) 70/30 PTT/PET. The arrow in (**f**) indicates the PTT fibril.

Figure 9 shows the atomic force microscopy (AFM) height images for the thin films of neat PTT, neat PET, and 70/30 PTT/PET obtained through isothermal melt crystallization at 215 °C. No clear structure was seen on the spherulite surface of the neat PET due to the thin fibrils in the spherulites (Figure 9a). Small undulations were seen in the height profile due to the presence of thin fibrils, i.e., the height of the undulation was about 30 nm, the width was about 100 nm, and the interfibrillar distance was about 200 nm (Figure 9b). In contrast, the fibrillar structure was clearly seen in the spherulite surface of neat PTT due to the presence of thick fibrils (Figure 9c). This large undulation was seen due to the presence of thick fibrils, i.e., the height of the undulation was about 50 nm, the width was about 500 nm, and the interfibrillar distance was about 1000 nm (Figure 9d). Due to their different fibrillar structures, the spherulite morphologies of neat PET and neat PTT also differed, as shown in Figure 1, i.e., the compact spherulites of the neat PET were composed of thin fibrils, whereas the dendritic spherulites of the neat PTT were composed of thick fibrils.

In the blend, both thin fibrils and thick fibrils were seen in the inner region (A in Figure 9e), while only thick fibrils were seen in the outer region (B in Figure 9e), indicating that the surface structures in the inner and outer regions differed. In the height profiles, both small and large undulations were seen in the height profiles in the inner region (A in Figure 9f), while only large undulations were seen in the outer region (B in Figure 9f). The thin fibrils with small undulations are attributed to PET fibrils, while the thick fibrils with large undulations are attributed to PTT fibrils. Thus, the different structures in the inner and outer regions of the spherulite are attributed to the double spherulite consisting of a compact PET spherulite composed of thin fibrils in the inner region and a dendritic PTT spherulite composed of thick fibrils in the outer region. The most significant results, shown in Figure 9e,f, are that the thick PTT fibrils are interfiled within the PET spherulite in the inner region and are arranged in the radial direction. The thick PTT fibrils interfiled within the PET spherulites could be attributed to the interfiling crystallization of the excluded PTT in the confined interfibrillar amorphous region within the PET spherulite. The interfiled PTT fibrils grown within the PET spherulite continued outside of the PET spherulite to the PTT spherulite in the outer region. Due to the continuous growth of the PTT fibrils from the inside of the PET spherulite in the inner region to the PTT spherulite in the outer region, the boundary between the PET spherulite and the PTT spherulite was unclear, although a double spherulite was formed, as shown in the polarized and optical micrographs in Figure 5e,f. These results suggest that the dendritic spherulite that emerged from the spherulite growth front of PET was due to the continuous growth of the PTT fibrils from the PET spherulite in the inner region to the PTT spherulite in the outer region after the spherulite growth of PET ceased.

### 3.6. Model for the Evolution of Double Spherulites

**Figure 10:**
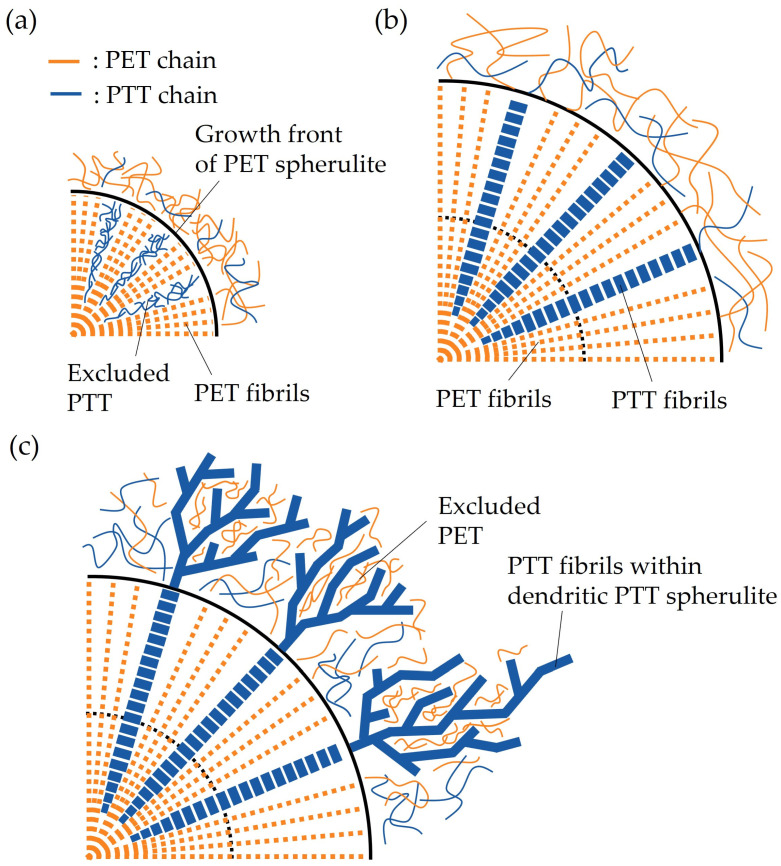
Schematic illustration of the evolution of a double spherulite in 70/30 PTT/PET: (**a**) exclusion of PTT into the interfibrillar amorphous region of PET within the PET spherulite; (**b**) interfiling crystallization in the confined interfibrillar amorphous region; and (**c**) continuous growth of PTT fibrils from the inside to the outside of the PET to form the dendritic PTT spherulite.

Figure 10 schematically shows the evolution of a double spherulite obtained through two-step crystallization in 70/30 PTT/PET during isothermal melt crystallization at 215 °C. The PET spherulite grew from the miscible melt of PTT and PET in the first crystallization step. During the spherulite growth of PET, PTT mixed with PET was excluded from the growth front of PET into the interfibrillar amorphous region within the PET spherulite (Figure 10a). The excluded PTT in the interfibrillar amorphous region crystallized and grew into long fibrils in the radial direction within the PET spherulite (Figure 10b). This reflects the interfiling crystallization of PTT in the confined interfibrillar amorphous region within the PET spherulite. The spherulite growth rate of PET gradually decreased as the concentration of the PTT at the growth front of PET increased. When the growth of the PET spherulite stopped, the PTT fibrils interfiled within the PET spherulite emerged from the growth front of the PET spherulite and continuously grew outward into the outer region to form a dendritic spherulite in the second crystallization step (Figure 10c). Due to the exclusion of PET into the interfibrillar amorphous region of PTT, the dendritic PTT spherulite was more open than that of the neat PTT. The characteristic two-step spherulite growth and the double spherulite formation are attributed to the following: (1) the exclusion of PTT into the interfibrillar amorphous region of PET during the spherulite growth of PET and interfiling crystallization of PTT in the confined interfibrillar amorphous region, which grew into fibrils in the radial direction within the PET spherulite; (2) the cessation of the spherulite growth of the PET due to the PTT being excluded at the spherulite growth front of PET; and (3) the continuous growth of the radially arranged PTT fibrils from inside the PET spherulite to outside the growth front of the PET spherulite to form the dendritic PTT spherulite.

## 4. Conclusions

We found that double spherulites consisting of a PET spherulite in the inner region and a PTT spherulite in the outer region could be obtained via the isothermal melt crystallization of 70/30 PTT/PET at 215 °C due to the two-step crystallization behavior, i.e., the compact PET spherulite grew in the first step, and then the dendritic PTT spherulite grew from the spherulite growth front of PET in the second step. Due to the two-step crystallization, the integrated Hv light-scattering intensity and the spherulite growth rate gradually increased with crystallization time in the first step and then steeply increased in the second step. The PET spherulite grew in the first step. The spherulite growth rate of PET decreased with time, and the decrease became larger as the interspherulite distance became shorter due to the excluded PTT at the growth front of the PET spherulite. The excluded PTT crystallized in the second step, as confirmed by the DSC measurements. AFM observation revealed that the radially arranged thick PTT fibrils were interfiled within the PET spherulite and continued to grow into the PTT spherulite in the outer region. Thus, the double spherulite formed due to the two-step crystallization behavior is attributed to (1) the exclusion of PTT into the amorphous region between PET fibrils and interfiling crystallization of PTT in the confined interfibrillar amorphous region within the PET spherulite; (2) the cessation of the spherulite growth of the PET due to the PTT excluded at the growth front; and (3) the continuous growth of radially arranged PTT fibrils from the inside of the PET spherulite to the outside of the PET spherulite to form a dendritic PTT spherulite. Exclusion behavior and interfiling crystallization could be helpful in understanding the origin of the double spherulites observed in other crystalline/crystalline polymer blends [32,33,34] and crystalline block copolymers [20,51,52,53], which is currently unclear. Understanding the evolution of double spherulites would provide insight into the basic concept of crystallization.

## Data Availability

Data are contained within the article.
